# Recombinant humanized type III collagen freeze-dried fibers for vaginal laxity: a narrative review of mechanisms, clinical evidence, and potential relevance to pelvic floor dysfunction

**DOI:** 10.3389/fmed.2026.1941555

**Published:** 2026-10-15

**Authors:** Runzhi Shi, Liehong Wang

**Affiliations:** 1 School of Clinical Medicine, Qinghai University, Xining, Qinghai, China; 2 Department of Obstetrics and Gynecology, Qinghai Red Cross Hospital, Xining, Qinghai, China

**Keywords:** collagen type III, pelvic floor disorders, regenerative medicine, tissue scaffolds, vaginal diseases

## Abstract

**Background:**

Vaginal laxity (VL) is a prevalent condition affecting women's quality of life, characterized by reduced vaginal tightness, sexual function impairment, and psychological distress. While surgical vaginoplasty and energy-based devices (radiofrequency and laser) are commonly used, these approaches have important limitations, including surgical risks, variable durability, and limited evidence quality. Collagen dysregulation is increasingly recognized as a key pathophysiological feature of VL, and collagen-based biomaterials have emerged as potential regenerative interventions. However, the evidence specifically linking collagen fiber biology to VL remains scattered, and a systematic synthesis of recombinant humanized type III collagen (rhCol III) for this indication is lacking.

**Objective:**

This narrative review systematically summarizes current evidence on the pathophysiological role of collagen in VL, the material properties and mechanisms of action of rhCol III freeze-dried fibers, and the available preclinical and clinical data for this intervention, with consideration of its potential relevance to the broader spectrum of pelvic floor dysfunction.

**Findings:**

Recombinant humanized type III collagen freeze-dried fibers exhibit good biocompatibility and biodegradability due to their structural homology with native human collagen. Their proposed mechanism of action involves three complementary pathways—physical support and structural filling, biochemical regeneration via upregulation of endogenous collagen synthesis, and microenvironment modulation including angiogenesis and anti-inflammatory effects—although several specific molecular pathways have been characterized primarily in non-vaginal tissue models and require direct validation in vaginal tissue. Available clinical evidence from a single small two-center randomized controlled trial (*n* = 84) demonstrates significant short-term improvements in VL symptoms and sexual function at 4 weeks, with a favorable short-term safety profile. However, the evidence base remains early-stage, with no large-scale multicenter trials, no long-term follow-up data, and no head-to-head comparisons with established treatments.

**Conclusion:**

rhCol III freeze-dried fibers represent an emerging, biologically plausible intervention for VL that warrants further investigation. Key knowledge gaps include the need for large-scale randomized trials with long-term efficacy and safety data, standardized treatment protocols, comparative effectiveness studies, and direct mechanistic validation in vaginal tissue. The potential applicability to other pelvic floor disorders remains speculative at this stage.

## 1 Introduction

Vaginal laxity (VL) is a common pelvic floor disorder that adversely affects women's physical and psychological well-being. It is primarily triggered by factors including pregnancy and childbirth, aging, menopause, obesity, and chronic elevation of intra-abdominal pressure—with pregnancy and delivery exerting the most profound impact on vaginal and pelvic floor anatomy (1, 5). Clinically, VL manifests as vaginal wall laxity, mucosal atrophy, diminished tissue elasticity, and weakened pelvic floor musculature (2, 3). These structural and functional alterations are frequently associated with sexual dysfunction, recurrent local inflammation, and other pelvic floor disorders (2, 3).

Despite its increasing prevalence—estimated to exceed current reported figures (5)—the diagnosis and treatment rates for VL remain suboptimal. Notably, over 80% of obstetricians and gynecologists report that patients underestimate the clinical significance of this condition (4). Current therapeutic strategies encompass both surgical and non-surgical modalities. Surgical intervention remains the standard of care for moderate-to-severe VL, whereas non-surgical approaches include radiofrequency therapy, pelvic floor rehabilitation, laser therapy, and electro-physiological stimulation (5–7). Among these, radiofrequency and laser therapies primarily act by inducing collagen fiber contraction and stimulating *de novo* collagen synthesis (6, 7).

Importantly, VL does not exist in isolation; it represents one clinical manifestation within the broader spectrum of pelvic floor dysfunction (PFD). The pelvic floor provides structural support not only for vaginal and uterine anatomy but also for the bladder and urethra, and its compromise frequently extends to lower urinary tract symptoms (LUTS). Epidemiological studies have demonstrated that women with VL exhibit a significantly elevated risk of concurrent stress urinary incontinence (SUI), with shared pathogenic mechanisms including collagen degradation, altered collagen subtype ratios, and disrupted extracellular matrix remodeling (11, 31, 32). The collagen-based pathological framework that underpins VL—particularly the deficiency and disorganization of type III collagen and the imbalance between matrix metalloproteinases (MMPs) and their tissue inhibitors (TIMPs)—is common to SUI, pelvic organ prolapse (POP), and other PFD subtypes (11, 15, 16). This pathogenic overlap provides a strong mechanistic rationale for evaluating collagen-targeted therapies, such as recombinant humanized collagen biomaterials, not only for VL but also for their potential applicability to the wider management of PFD and associated LUTS (31, 33).

Pathologically, VL is characterized by degradation of elastic fibers, aberrant collagen remodeling, reduced total collagen content in the vaginal wall, and disorganized collagen fiber alignment—collectively resulting in compromised tissue resilience and elasticity (8). Emerging evidence indicates that vaginal tissue from VL patients exhibits significantly decreased collagen protein levels, suggesting that collagen supplementation may represent a viable therapeutic avenue (9). Given its biocompatibility, low immunogenicity, and intrinsic role in extracellular matrix integrity, collagen is considered an ideal biomaterial for tissue repair in this context (10, 22).

Despite the well-documented role of collagen dysregulation in pelvic floor disorders, the existing literature exhibits two notable gaps. First, mechanistic investigations of collagen remodeling have been conducted predominantly in the context of pelvic organ prolapse (POP), whereas collagen-specific pathophysiology in vaginal laxity (VL) remains comparatively underexplored. Second, prior reviews on VL have largely focused on clinical diagnosis and existing treatment modalities, with limited attention to the collagen-based biomaterial approaches that are emerging as potential regenerative therapies. Concurrently, recombinant humanized type III collagen (rhCol III) has shown promising regenerative properties in dermatology, wound healing, and other tissue engineering applications; however, its specific application to vaginal tissue repair has only recently begun to be investigated, and the evidence base remains scattered across preclinical and early clinical studies.

Recombinant humanized type III collagen freeze-dried fibers—produced via advanced genetic engineering—exhibit structural homology to native human type III collagen (20). Their freeze-dried fibrous architecture provides a three-dimensional scaffold for mucosal regeneration, with emerging evidence from preclinical and early clinical studies supporting potential efficacy in vaginal mucosal repair (10, 23). Building on these developments, the present narrative review aims to synthesize current evidence across three domains: (1) the collagen-mediated pathophysiology of VL and its mechanistic overlap with broader pelvic floor dysfunction; (2) the material properties and mechanisms of action of rhCol III freeze-dried fibers; and (3) the available preclinical and clinical evidence for this approach, including its safety profile. By integrating mechanistic insights with emerging clinical data, this review seeks to clarify what has been established specifically for VL, what remains extrapolated from other tissue contexts, and where future research is most urgently needed.

## 2 Collagen alterations in the pathophysiology of vaginal laxity and pelvic floor dysfunction

VL is characterized by collagen-related pathological alterations, including reduced collagen content in the vaginal wall and pelvic floor supportive tissues; an imbalanced ratio of type I to type III collagen; structural and organizational abnormalities of collagen fibers; dysregulation of collagen degradation and synthesis; and hormone-driven collagen degeneration—collectively resulting in diminished tissue tensile strength and elasticity (11, 31). These same collagen-mediated pathologies are central to the development of SUI and POP, underscoring the shared mechanistic basis between VL and other PFD subtypes (31, 32).

The collagen-mediated pathological changes underlying VL and its overlap with other PFD subtypes involve complex alterations across multiple tissue layers and molecular pathways. To provide a visual framework for these changes, Figure 1 summarizes the structural organization of the normal vaginal wall, the key ECM remodeling events that occur in VL, and the overall therapeutic rationale for collagen-based intervention.

**Figure 1:**
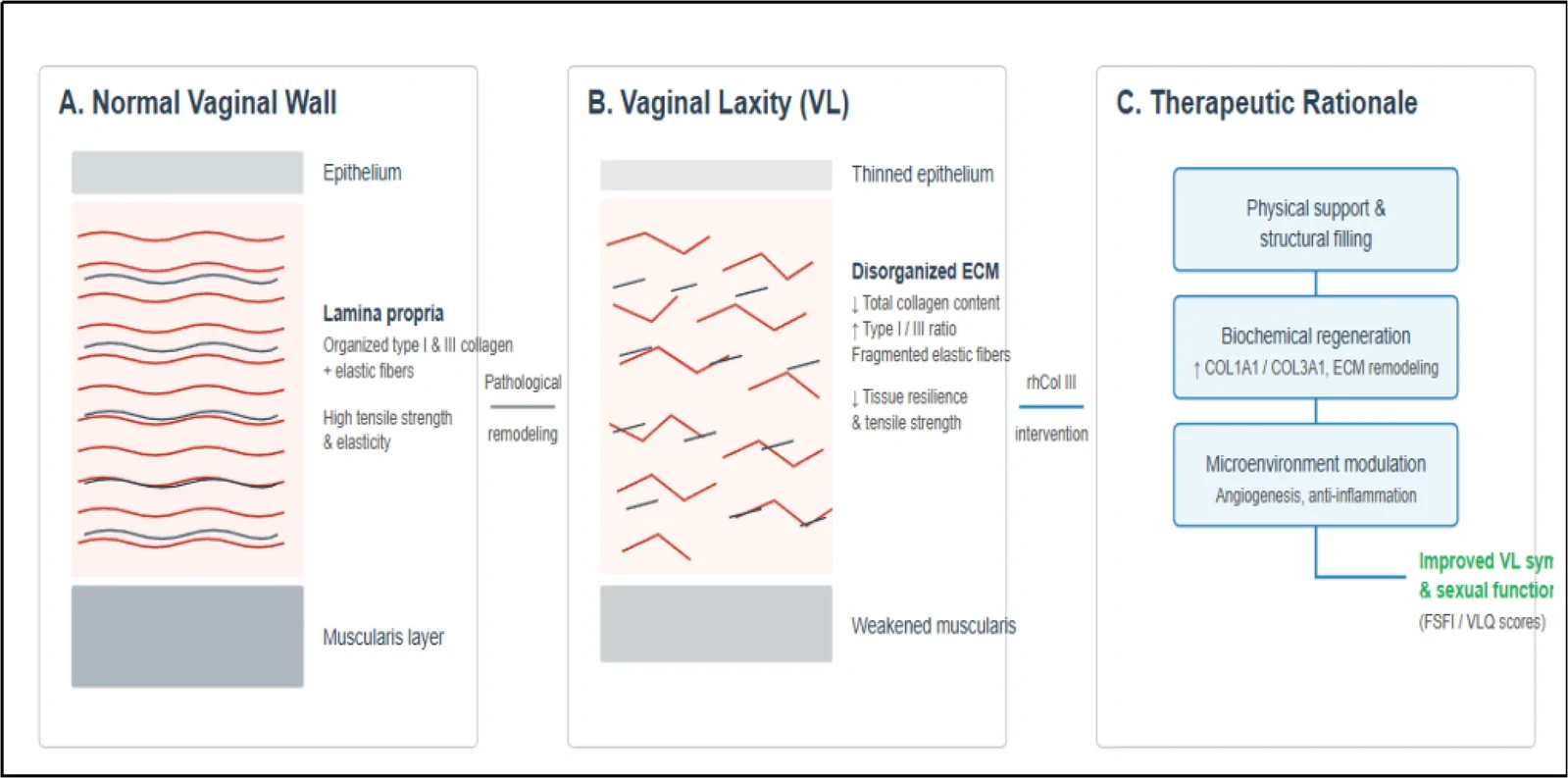
Schematic overview of vaginal extracellular matrix remodeling in vaginal laxity and the collagen-based therapeutic rationale. **(A)** Normal vaginal wall showing the organized three-layer structure: the stratified squamous epithelium, the lamina propria (rich in organized type I and type III collagen fibers and elastic fibers, providing tensile strength and elasticity), and the underlying muscularis layer. **(B)** Vaginal laxity (VL) is characterized by pathological extracellular matrix remodeling, including epithelial thinning, disorganized and reduced collagen content with an elevated type I/III collagen ratio, fragmented elastic fibers, and weakened muscular support. These structural changes underlie the loss of tissue resilience and tensile strength characteristic of VL. **(C)** Collagen-based therapies such as recombinant humanized type III collagen (rhCol III) freeze-dried fibers target these pathological alterations through three complementary modes—physical support, biochemical regeneration, and microenvironment modulation—ultimately leading to improved VL symptoms and sexual function (measured by FSFI and VLQ scores). ECM, extracellular matrix; VL, vaginal laxity; rhCol III, recombinant humanized type III collagen; FSFI, Female Sexual Function Index; VLQ, Vaginal Laxity Questionnaire.

### 2.1 Abnormal collagen content and subtype ratio

Previous studies have indicated that a reduction in total collagen content was observed in the vaginal wall, pubocervical fascia, and uterosacral ligaments of patients with VL, accompanied by diminished tissue support capacity and tensile strength (11). This work established that the underlying pathology of VL involves decreased collagen content, an imbalanced ratio of type I to type III collagen, and disruption of elastic fibers (11, 14). Notably, similar alterations—including decreased total collagen and an elevated type I/III ratio—have been consistently documented in the urethral and periurethral connective tissues of women with SUI, suggesting a common collagenopathy across PFD manifestations (32). Some scholars have further proposed that abnormal collagen remodeling and excessive elongation of the levator ani muscle constitute key pathological features of VL (12, 13). Others suggested that the synergistic degeneration of collagen and elastic fibers represents a critical mechanism underlying the loss of vaginal wall support (8, 14).

### 2.2 Collagen degeneration–synthesis imbalance

Some studies have reported that collagen III, tissue inhibitor of metalloproteinase-1 (TIMP- 1), and transforming growth factor-beta 1 (TGF-β1) are downregulated in the vaginal wall tissues of patients with pelvic organ prolapse (POP), and their expression levels correlate with disease severity—suggesting potential involvement in the pathogenesis and progression of POP (15, 16). Moreover, significant correlations exist among collagen III, TIMP-1, and TGF-β1 in the vaginal wall tissues of POP patients, indicating a degree of functional synergy among these molecules in disease development (15). Alterations in the collagen type ratio within the vaginal wall are accompanied by corresponding changes in collagen ultrastructure (11, 16).

Additionally, collagen proteins—particularly types I and III—play critical structural roles in the pelvic floor and vaginal supportive tissues, thereby contributing essential mechanical support for maintaining the normal anatomical position of pelvic organs. Among collagen-degrading enzymes, matrix metalloproteinase-1 (MMP-1) is a major protease responsible for the degradation of both type I and type III collagen. Its enzymatic activity is primarily regulated by TIMP-1, and the dynamic interplay between MMP-1 and TIMP- 1 is crucial for maintaining homeostasis in collagen turnover (15). Xue et al. demonstrated that TGF-β1 cooperatively suppresses the β-catenin signaling pathway, leading to reduced collagen synthesis and enhanced collagen degradation (17). Importantly, this MMP/TIMP imbalance has also been identified in the urethral sphincter complex of patients with SUI, further supporting the concept of a unified collagen-degradation pathway across PFD subtypes (32, 33). Collectively, dysregulation of these molecular mediators disrupts the balance between collagen degradation and synthesis—a key collagen-related pathological mechanism underlying vaginal laxity and its associated pelvic floor disorders.

### 2.3 Effects of estrogen on collagen

A decline in estrogen levels contributes to the progression of vaginal laxity. As one of the primary sex hormones in females, estrogen promotes angiogenesis, enhances blood flow, modulates inflammatory cytokines, improves tissue compliance, and regulates collagen content (18, 19). The postmenopausal decline in estrogen not only accelerates vaginal mucosal atrophy and collagen loss in the vaginal wall but also has well-documented effects on the lower urinary tract, including urethral mucosal thinning, decreased urethral closure pressure, and increased susceptibility to SUI (40). Estrogen receptors are densely expressed in vaginal, urethral, and bladder tissues, and hormonal withdrawal triggers concurrent deterioration of collagen content and organizational architecture across all these pelvic floor compartments—providing further evidence that VL and LUTS share a common hormone-sensitive, collagen-dependent pathophysiology (18, 40).

### 2.4 Translational implications for broader pelvic floor dysfunction

The collagen-mediated pathophysiological changes described above are not unique to VL; similar alterations in collagen content, subtype ratio, and MMP/TIMP balance have been documented in other pelvic floor disorders, particularly stress urinary incontinence (SUI) and pelvic organ prolapse (POP). This shared pathogenic framework provides a theoretical rationale for considering collagen-targeted therapies—such as rhCol III freeze-dried fibers—across the broader PFD spectrum.

However, several important caveats must be emphasized. First, while the mechanistic overlap is well established, the specific contribution of collagen dysregulation may differ across PFD subtypes: for example, SUI involves urethral sphincter dysfunction as well as connective tissue weakness, and POP involves multi-compartmental defects that may not be fully addressed by collagen supplementation alone. Second, the anatomical site of delivery and the mechanical requirements differ substantially between the vaginal submucosa and the urethral or pelvic supportive tissues. Third, clinical trials of rhCol III specifically for SUI or POP have not yet been conducted, and any extrapolation from VL evidence remains speculative at this stage.

Preclinical evidence from recent studies provides preliminary support for the collagen-regenerative approach in PFD. Specifically, Zhang et al. demonstrated that rhCol III promotes extracellular matrix remodeling in a rat model of simulated birth injury by restoring fibroblast function and stimulating angiogenesis, including upregulation of COL1A1, COL3A1, CD31, CD34, and VEGFA (14). Another study by Zhang et al. showed enhanced fibroblast function in POP tissue-derived cells (28). However, further preclinical and early-phase clinical studies are needed before clinical translation can be considered.

## 3 Material properties of recombinant humanized type III collagen freeze-dried fibers

Recombinant humanized type III collagen, whose amino acid sequence matches that of the functional domain of native human type III collagen, forms reticular fibers adopting a triple-helical conformation with a helical angle of 164.88° (20). Owing to its good biocompatibility and biological activity, recombinant human collagen is an important biomaterial in the field of tissue engineering.

### 3.1 Humanized structure with high biocompatibility and biodegradability

Collagen-based biomaterials can be categorized along two primary axes: collagen type and source. With respect to collagen type, type I collagen is the most abundant structural collagen in connective tissues and provides high tensile strength, whereas type III collagen is the principal collagen in reticular fibers and embryonic tissues, contributing to tissue elasticity and wound healing (22). In the vaginal wall, both type I and type III collagen are present, with type III collagen playing a particularly important role in maintaining tissue distensibility and elasticity—properties that are critical to vaginal function. Other collagen types (e.g., types IV, V, and VII) contribute to basement membrane integrity and fiber assembly but are not typically used as primary structural biomaterials for soft tissue augmentation. In the context of vaginal laxity, type III collagen is of particular therapeutic interest because its relative deficiency and disorganization are well documented in affected tissues, and its replenishment may directly address the specific collagen subtype imbalance observed in the condition (11, 14).

With respect to source, collagen used in biomedical applications is predominantly derived from animal tissues (bovine, porcine, or marine), which offers low cost and abundant supply but carries several notable limitations. These include batch-to-batch variability due to differences in source tissue and extraction methods, potential immunogenicity from xenogeneic epitopes (e.g., the α-Gal epitope in non-primate mammals), and the risk of pathogen transmission (e.g., prions, zoonotic viruses) (21). Recombinant humanized collagen, produced via recombinant DNA technology in expression systems such as yeast, bacteria, or mammalian cells, addresses many of these limitations: it contains no xenogeneic protein contaminants, exhibits consistently low immunogenicity, eliminates the risk of animal-derived pathogens, and offers high batch-to-batch reproducibility (20, 22). However, recombinant production also has drawbacks: it is generally more expensive than animal-derived collagen, yields can be lower, and the recombinant protein may require additional processing to achieve proper triple-helix folding and native-like bioactivity.

Recombinant humanized type III collagen freeze-dried fibers combine the theoretical advantages of type III collagen subtype specificity with the safety and reproducibility of recombinant production. These fibers contain no xenogeneic contaminants and demonstrate favorable biocompatibility and biodegradability, supporting their application in tissue engineering, wound management, and mucosal repair (22, 23). Nevertheless, it should be acknowledged that the long-term *in vivo* performance of recombinant humanized type III collagen in vaginal tissue—including its degradation kinetics, host tissue integration, and sustained bioactivity—has not yet been fully characterized, and direct head-to-head comparisons with animal-derived collagen products in this specific anatomical context remain limited.

### 3.2 Porous structure of lyophilized fibers

Recombinant humanized type III collagen lyophilized fibers exhibit a loose, porous microfibrous morphology. Upon implantation, they rapidly absorb water and swell, while their mechanical strength can be tuned to match that of native soft tissues—supporting good conformity to the vaginal wall and providing structural support and mechanical reinforcement (23). As reported by some scholars, these lyophilized fibers can be engineered into a highly porous, biodegradable scaffold that facilitates cellular infiltration and host tissue ingrowth. Critically, the degradation kinetics of the scaffold are synchronized with the host's tissue remodeling process: it delivers transient mechanical support while concurrently promoting *de novo* collagen deposition, thereby enabling functional tissue integration and reducing the risk of permanent foreign-body retention (24).

### 3.3 Biologically active induction of regeneration

Recombinant humanized type III collagen stimulates endogenous collagen production and promotes the synthesis of endogenous types I and III collagen. Lin-Hui et al. reported that recombinant humanized type III collagen can be internalized by fibroblasts and significantly upregulates the expression of endogenous collagen-encoding genes—namely, COL1A1 (encoding type I collagen) and COL3A1 (encoding type III collagen) (26). This mechanism drives *de novo* collagen synthesis, shifting the tissue repair paradigm from passive filler-based restoration to active, biologically driven regeneration. Furthermore, it restores the physiological type I/type III collagen ratio in damaged connective tissues.

### 3.4 Strong tissue integration

Recombinant humanized type III collagen exhibits high biointegration with the host tissue, leaving no foreign-body residue, and serves as a supportive scaffold for cell migration and proliferation. Chen et al. reported that recombinant humanized type III collagen microgels demonstrate progressive *in vivo* biodegradation, achieving near-complete absorption within six weeks post-injection (25). This degradation process occurs concurrently with seamless integration into the host's dermal tissue, with no observable foreign-body reaction or residual material. Histological analysis confirmed robust neotissue formation at the implantation site, and the degraded scaffold was fully replaced by endogenous, host-derived extracellular matrix (ECM).

### 3.5 Rationale for the freeze-dried fiber formulation and comparison with alternative collagen delivery formats

The choice of freeze-dried fibers as a delivery format for rhCol III is motivated by a combination of structural, mechanical, and practical considerations, and it is important to situate this formulation within the broader landscape of collagen-based biomaterial formats used in soft tissue repair. Alternative collagen formulations include hydrogels, sponges, films, microspheres, and injectable solutions, each with distinct advantages and limitations depending on the clinical context.

Hydrogels are highly hydrated, injectable, and conform well to irregular tissue defects, making them suitable for filling irregular cavities and delivering cells or growth factors (24). However, their mechanical strength is generally low, and they may degrade rapidly *in vivo*, limiting their utility for applications requiring sustained structural support—such as vaginal wall reinforcement. Collagen sponges, produced by freeze-drying but typically in a three-dimensional block form, offer high porosity and good cell infiltration but are less amenable to minimally invasive delivery and may not uniformly distribute within narrow tissue planes (24). Collagen films provide a barrier function and mechanical integrity but are primarily used for guided tissue regeneration or wound coverage rather than volumetric augmentation. Injectable collagen solutions (e.g., dermal fillers) offer ease of delivery but have limited structural persistence and are rapidly resorbed.

Freeze-dried fibers occupy a distinctive niche among these formats. Compared with hydrogels and injectable solutions, the fibrous architecture provides greater initial mechanical support and structural integrity after implantation, which is relevant for countering the tissue laxity characteristic of VL. Compared with sponges and films, freeze-dried fibers can be delivered via minimally invasive injection or cannula-based placement, allowing uniform distribution within the submucosal and fascial layers of the vaginal wall (23, 27). The lyophilization process preserves the native triple-helix conformation of collagen, maintaining bioactivity while conferring long shelf stability without the need for refrigeration—a practical advantage for clinical use. Additionally, the porous microfibrillar structure supports fibroblast attachment, migration, and endogenous tissue ingrowth, enabling a transition from passive filling to active tissue regeneration over time (23, 24).

Despite these theoretical advantages, the freeze-dried fiber formulation also has limitations. The lyophilized structure may be fragile and prone to fragmentation during handling or delivery. Hydration and swelling kinetics *in vivo* can be difficult to predict, potentially affecting the precision of volumetric correction. Furthermore, the degradation rate and mechanical persistence of the fibrous scaffold in the vaginal microenvironment—where there is continuous exposure to moisture, mechanical strain, and a rich microbial community—have not been fully characterized. Whether the fiber format offers meaningful clinical advantages over, for example, well-standardized collagen hydrogels or cross-linked collagen fillers in the specific context of VL remains an open question that will require direct comparative studies.

## 4 Mechanism of action of rhCol III lyophilized fibers in VL treatment

The therapeutic action of rhCol III freeze-dried fibers operates through a multimodal mechanism encompassing physical support, biochemical regeneration, and local microenvironment modulation.

Building on the material properties described above, rhCol III freeze-dried fibers exert their therapeutic effects on VL through a multimodal mechanism that integrates physical, biochemical, and microenvironmental pathways. Figure 2 provides a schematic overview of these three complementary mechanisms, with color coding to distinguish between directly demonstrated effects and those extrapolated from other tissue models.

**Figure 2:**
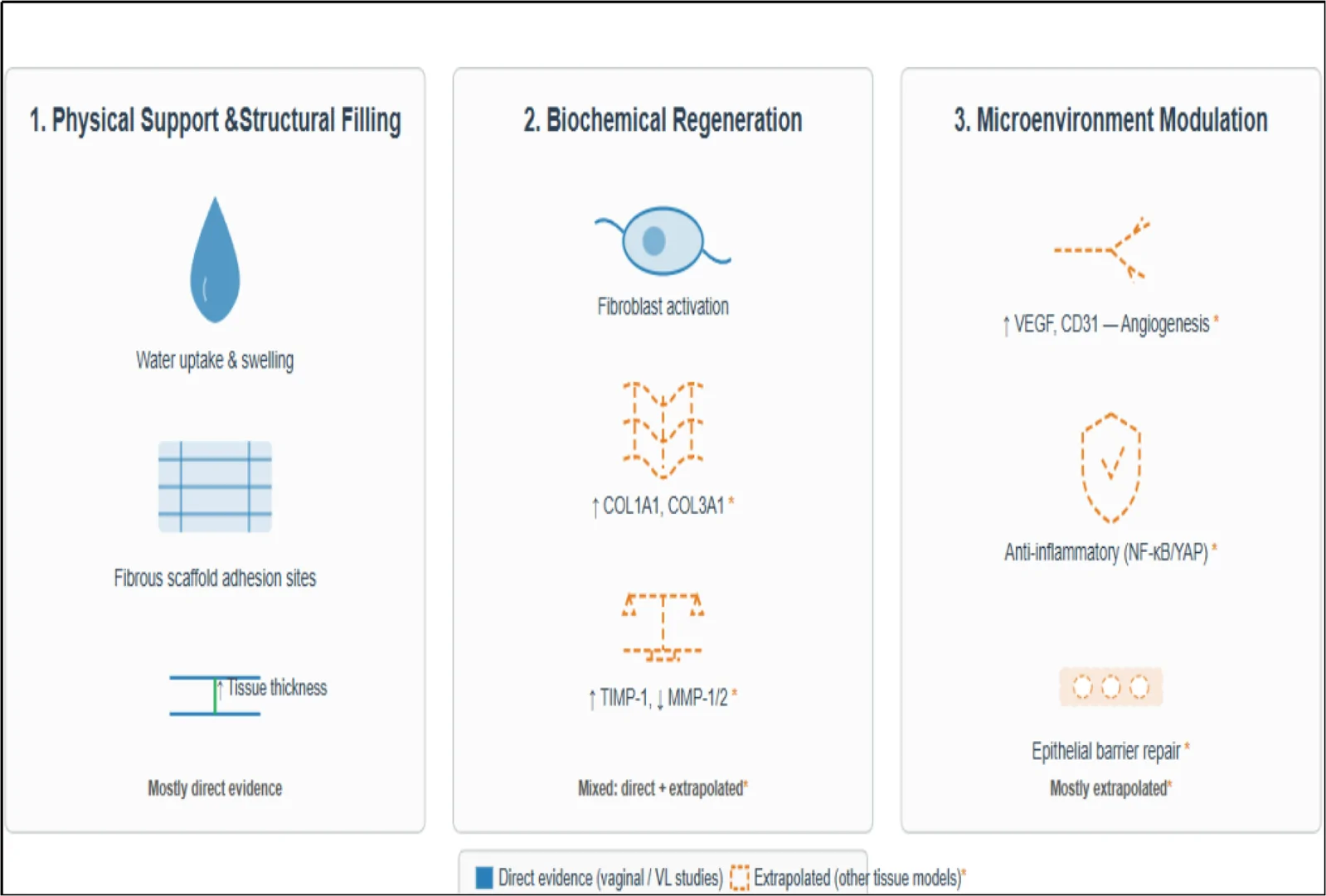
Proposed mechanisms of recombinant humanized type III collagen freeze-dried fibers in vaginal laxity treatment. The therapeutic action operates through three complementary pathways: (1) Physical support and structural filling, providing immediate biomechanical reinforcement via water uptake, fibrous scaffold adhesion, and increased tissue thickness; (2) Biochemical regeneration, activating fibroblast-mediated endogenous collagen synthesis (upregulation of COL1A1 and COL3A1) and restoring the collagenase/anti-collagenase balance (↑ TIMP-1, ↓ MMP-1/2); and (3) Microenvironment modulation, enhancing angiogenesis (↑ VEGF, CD31), exerting anti-inflammatory effects (via the NF-κB/YAP pathway), and promoting epithelial barrier repair. Color coding indicates evidence strength: solid blue elements represent mechanisms supported by direct evidence from vaginal/VL studies or by intrinsic material properties of collagen fibers; elements marked with an asterisk (*) and rendered in dashed orange represent mechanisms extrapolated from non-vaginal tissue models (e.g., skin scar, endometrium) that have not yet been directly validated in vaginal tissue. COL1A1, collagen type I alpha 1 chain; COL3A1, collagen type III alpha 1 chain; MMP, matrix metalloproteinase; TIMP, tissue inhibitor of metalloproteinase; VEGF, vascular endothelial growth factor; NF-κB, nuclear factor kappa-light-chain-enhancer of activated B cells.

### 4.1 Physical support and structural filling

rhCol III lyophilized fibers rapidly fill the submucosal layer and fascial spaces upon implantation, immediately increasing vaginal wall thickness and tissue tautness—thereby compensating for collagen loss associated with vaginal laxity. Moreover, the lyophilized fibers preserve the native collagen triple-helix conformation, forming a biomimetic scaffold that provides adhesion sites for fibroblasts and epithelial cells, thereby guiding their orderly migration and proliferation (27). Concurrently, physical crosslinking between fibers enhances the tensile strength and elastic modulus of the vaginal wall, directly ameliorating the biomechanical manifestations of laxity.

### 4.2 Activation of fibroblasts to promote collagen regeneration

Recombinant humanized type III collagen promotes proliferation and migration of vaginal fibroblasts, inhibits cellular senescence, and upregulates the expression of COL1A1 and COL3A1, thereby enhancing endogenous synthesis of types I and III collagen. It also upregulates TIMP- 1 while suppressing MMP-1 and MMP-2, thereby reducing collagen degradation and facilitating reconstruction of the collagen network (26). Furthermore, it induces elastin secretion, enabling the formation of a dense, well-organized elastic fiber network in concert with newly synthesized collagen—ultimately restoring vaginal wall elasticity and recoil capacity (27).

### 4.3 Improving the local microenvironment to facilitate tissue repair

rhCol III upregulates VEGF, CD31, and CD34 expression, thereby increasing microvessel density in the vaginal wall, improving local blood supply and nutrient delivery, accelerating tissue repair, and promoting proliferation and differentiation of vaginal epithelial cells—ultimately leading to thickening of the mucosal layer and restoration of vaginal rugae, as well as enhanced lubrication and improved vaginal microbiota homeostasis (28). Concurrently, it modulates cellular metabolism and attenuates inflammatory responses by inhibiting the NF-κB/YAP signaling pathway, thereby reducing infection risk (27).

## 5 Advances in clinical research

### 5.1 Efficacy

In 2024, Chen et al. reported findings from the world's first prospective, randomized, two-center clinical trial specifically evaluating rhCol III freeze-dried fibers for the treatment of vaginal laxity (30). A total of 84 women with VL were enrolled across two centers in China and randomly assigned to either the study group (42 patients, treated with rhCol III freeze-dried fibers in combination with a medical type III collagen vaginal gel used as the delivery vehicle) or the control group (42 patients, treated with the medical type III collagen vaginal gel alone). At the 4-week post-treatment assessment, the quantitative improvement rate in vaginal laxity grading scores was 88.10% (37/42) in the study group—significantly higher than the 30.95% (13/42) observed in the control group (*P* < 0.001). After accounting for the effect of the collagen gel delivery vehicle, the independent efficacy rate of rhCol III freeze-dried fibers was estimated at 57.14%, indicating a direct therapeutic contribution of the fibrous scaffold beyond the gel formulation alone.

The study group also demonstrated significantly greater improvements than the control group in both the total Female Sexual Function Index (FSFI) score and the Vaginal Laxity Questionnaire (VLQ) score (*P* < 0.05). The most pronounced enhancements were observed across four domains: vaginal lubrication during sexual activity, alleviation of dyspareunia, satisfaction with sexual life, and subjective sexual arousal capacity. No significant between-group difference was observed in the rate of adverse events, and no serious adverse events were reported in either group.

Several important contextual considerations should be borne in mind when interpreting these findings. First, the comparator was a medical type III collagen vaginal gel rather than an established active treatment such as radiofrequency or laser therapy, so the relative efficacy of rhCol III fibers compared with energy-based modalities remains unknown. Second, the follow-up duration was only 4 weeks, and the durability of the therapeutic effect beyond this time point has not been established. Third, the study was conducted exclusively in a Chinese population, and generalizability to other ethnic or age groups cannot be assumed. Fourth, while the study was described as a randomized controlled trial, the published report provides limited details regarding allocation concealment, blinding procedures, and intention-to-treat analysis, which limits formal risk-of-bias assessment. Fifth, the use of a collagen gel as both the delivery vehicle in the intervention arm and as the sole treatment in the control arm means that the study design isolates the incremental effect of the freeze-dried fiber format rather than the effect of collagen *per se*—an important distinction for understanding the mechanism of action. Despite these limitations, this trial represents an important first step in the clinical evaluation of rhCol III freeze-dried fibers for VL and provides a foundation for larger, more rigorous comparative studies.

You et al. further corroborated the efficacy of rhCol III freeze-dried fibers administered via local intravaginal delivery for treating vaginal atrophy and laxity (10). Their study confirmed that rhCol III promotes restoration of the vaginal epithelial barrier and enhances vaginal tissue elasticity, while also establishing its favorable biocompatibility and biological activity. These findings provide a solid scientific foundation for the continued clinical development of rhCol III freeze-dried fibers and the expansion of its therapeutic indications.

More recently, Zhang et al. demonstrated that recombinant humanized collagen enhanced fibroblast function and angiogenesis in a pelvic organ prolapse model, providing additional preclinical support for the application of this biomaterial across the broader spectrum of pelvic floor disorders (28).

### 5.2 Safety

The safety profile of exogenous collagen biomaterials is determined by multiple interacting factors, including the source and type of collagen, structural and physicochemical properties, processing methods, delivery route, and the biological characteristics of the host tissue environment. In the context of vaginal application, safety considerations are further shaped by the unique features of the vaginal microenvironment—including a moist mucosal surface, a commensal microbial ecosystem, constant mechanical strain from pelvic floor activity, and proximity to the urethra and rectum.

#### 5.2.1 Immunogenicity: recombinant humanized vs. animal-derived collagen

Immunogenicity remains one of the most critical safety distinctions between recombinant humanized collagen and animal-derived collagen products. Animal-derived collagens—particularly those from bovine and porcine sources—carry xenogeneic epitopes that can trigger immune responses in human recipients. The best-characterized of these is the galactose-α-1,3-galactose (α-Gal) epitope, which is expressed in most non-primate mammals but absent in humans (21, 29). Preformed anti-α-Gal antibodies in human serum can mediate hypersensitivity reactions, and repeated exposure may boost antibody titers. Additionally, animal-derived collagen preparations may contain trace amounts of non-collagenous proteins, lipids, or cellular remnants that can serve as additional antigens, contributing to batch-to-batch variability in immunogenic potential.

Recombinant humanized collagen, by contrast, is produced using human collagen gene sequences in non-animal expression systems (e.g., yeast or bacterial hosts), and thus lacks α-Gal and other xenogeneic epitopes (20, 22). Clinical studies of recombinant human collagen used in dermal fillers and wound care have reported low rates of adverse immune reactions, generally limited to mild, transient local reactions (25, 29). However, several important caveats must be noted. First, the expression host system itself may introduce impurities—such as residual host cell proteins or endotoxins—that could trigger innate immune responses if not rigorously removed during purification. Second, while the amino acid sequence matches human collagen, differences in post-translational modifications (e.g., hydroxylation, glycosylation) between the expression system and native human tissue could theoretically affect antigenicity or bioactivity, although the clinical significance of this remains uncertain. Third, long-term immunological data—particularly with repeated administrations in the vaginal compartment—are currently lacking (42).

#### 5.2.2 Inflammatory and foreign-body responses

Implantation of any exogenous biomaterial, including collagen, triggers an initial host response that encompasses acute inflammation, macrophage recruitment, and the foreign-body reaction cascade. The nature and duration of this response depend on the material's physicochemical properties—including particle size, fiber morphology, surface chemistry, degradation rate, and the degree of cross-linking (24). Generally, highly porous, biodegradable scaffolds with degradation kinetics synchronized to tissue repair are associated with a milder and more transient inflammatory response.

In the case of rhCol III freeze-dried fibers, the porous fibrous architecture and non-cross-linked native-like structure are expected to facilitate gradual biodegradation and integration with host tissue, potentially minimizing chronic foreign-body reaction. Preclinical studies of recombinant human collagen microgels and scaffolds have demonstrated progressive replacement by endogenous ECM with minimal fibrous encapsulation (25, 27). However, it is important to distinguish between the biological effects of the collagen molecule itself and the physical attributes of the fiber formulation. Collagen as a biological signal is generally anti-inflammatory and promotes constructive tissue remodeling; conversely, the physical presence of a fibrous scaffold—particularly if degradation is too slow or if particles fragment—could theoretically sustain a chronic low-grade inflammatory response or induce foreign-body giant cell formation. Whether this occurs in the vaginal microenvironment, where mechanical loading and moisture exposure may accelerate scaffold breakdown, is not yet known.

Furthermore, the vaginal mucosa is an immunologically active tissue with a dense population of antigen-presenting cells and a tightly regulated mucosal immune system. The introduction of exogenous biomaterials into this environment may modulate local immune homeostasis in ways that are not fully predictable from studies in skin or other tissues. Local complications such as redness, swelling, or discharge have been reported with other intravaginal collagen-based products, although these are typically mild and self-limiting (10, 30). Systemic adverse events appear to be rare.

#### 5.2.3 Degradation products and metabolic fate

The safety profile of collagen biomaterials also depends on the nature and clearance of their degradation products. Native collagen is degraded by matrix metalloproteinases (MMPs) and other proteases into small peptide fragments and free amino acids, which are subsequently reutilized in physiological collagen turnover or cleared via normal metabolic pathways. This natural degradation pathway is generally considered biologically benign.

However, several factors can modify the safety of degradation products. First, if the collagen scaffold contains chemical cross-linking agents (e.g., glutaraldehyde, genipin) to enhance stability, the release of cross-linker residues during degradation may introduce cytotoxic or immunogenic effects. Recombinant humanized type III collagen freeze-dried fibers, in their native non-cross-linked form, would not carry this risk; however, if cross-linked variants are developed in the future, this would require careful safety assessment. Second, the rate of degradation relative to the rate of tissue replacement matters: excessively rapid degradation can lead to loss of structural support and release of large amounts of peptide fragments that may overwhelm local clearance mechanisms, whereas excessively slow degradation can promote chronic inflammation. Third, the potential for degradation products to influence local cellular behavior—for example, collagen-derived matrikines with signaling activity—should be considered, as these could have either beneficial or undesirable biological effects depending on concentration and context.

#### 5.2.4 Additional safety considerations for vaginal application

Beyond immunogenicity and degradation, several additional safety considerations are specific to the vaginal application of collagen biomaterials. The vaginal microbiome plays a critical role in mucosal defense, and the introduction of an exogenous biomaterial could potentially alter microbial composition, although the clinical significance of this is unknown. Infection risk, while low with proper aseptic technique, remains a theoretical concern, particularly given the proximity of the vaginal canal to the perineum and gastrointestinal tract. Mechanical complications such as migration, extrusion, or uneven distribution of the implanted material have been reported with other injectable bulking agents used in the pelvic floor and would need to be monitored in clinical trials of rhCol III fibers (34).

Finally, it should be emphasized that the current safety database for rhCol III freeze-dried fibers specifically in vaginal application is very limited—derived primarily from a single randomized controlled trial with only four weeks of follow-up (30)—and that longer-term safety data, including data on repeated treatments, pregnancy-related outcomes, and effects on the vaginal microbiome, are not yet available.

### 5.3 Positioning within the current therapeutic landscape

To contextualize the emerging evidence for rhCol III freeze-dried fibers, it is important to consider its position relative to existing treatment modalities for VL. Surgical vaginoplasty remains the gold standard for moderate-to-severe cases, offering long-term efficacy but with higher risk and longer recovery (2). Non-surgical options such as radiofrequency and laser therapy are widely used, with moderate short-term efficacy supported by multiple prospective studies and at least one RCT (5, 43), but concerns remain regarding durability and long-term safety (6, 7). Pelvic floor muscle training, as a first-line conservative approach, is safe and well-established but has limited efficacy for structural laxity and requires sustained adherence (37, 38). See Table 1.

**Table 1:** Comparative overview of treatment modalities for vaginal laxity.

Modality	Mechanism of action	Invasiveness	Efficacy duration	Recovery time	Key limitations	Evidence level
Surgical vaginoplasty	Surgical tightening of vaginal muscles and perineal body (2)	High	Long-term (2)	4–8 weeks (2)	Risk of scarring, dyspareunia, and surgical complications; not suitable for mild cases (2)	Level: Well-established.
Radiofrequency	Thermal collagen contraction and neocollagenesis via controlled heating (6, 36)	Low	6–12 Months;variable beyond that (5, 6, 43)	None	Limited durability; requires repeated sessions; inconsistent long-term evidence (6, 36)	Level: Moderately established.
Laser therapy	Thermal remodeling of vaginal mucosa via laser-induced microablative and non-ablative effects (7, 35)	Low	6–12 Months, variable (7, 35)	None	Limited high-quality evidence; potential mucosal damage; expensive equipment (7, 35)	Level: Moderately established, but controversial.
Pelvic floor muscle training	Voluntary contraction and strengthening of levator ani and pelvic floor muscles (38)	None	Variable; Requires ongoing adherence (38)	Ongoing	Low patient compliance; limited efficacy for structural mucosal laxity (38)	Level: Well-established as first-line conservative therapy.
rhCol III freeze-dried fibers	Collagen replenishment, endogenous regeneration via COL1A1/COL3A1 upregulation, and microenvironment modulation (26, 27, 30)*	Low(minimally invasive)	TBD (>4 weeks sustained in initial trial) (30)	Minimal	Early-stage evidence; no standardized treatment protocols; long-term safety data lacking; only one small RCT available; injection-related complications (e.g., migration, extrusion) reported with other bulking agents in pelvic floor applications (30, 34, 44)	Level: Early/Investigational.

TBD, to be determined; rhCol III, recombinant humanized type III collagen.

* Note on mechanistic references for rhCol III: Reference (30) provides direct clinical evidence from a vaginal laxity trial. References (26, 27) describe mechanisms demonstrated in non-vaginal tissue models (hypertrophic scar and endometrial immune microenvironment, respectively) and are extrapolated to vaginal tissue; direct validation of these specific molecular pathways in vaginal tissue remains limited.

In this landscape, rhCol III freeze-dried fibers occupy a distinctive niche as a minimally invasive, biologically active intervention that combines immediate structural filling with endogenous regenerative stimulation. However, it is critical to acknowledge that the current evidence base for rhCol III—comprising only one small two-center RCT with 4-week follow-up (30)—is substantially less robust than that for established treatments. Whether the dual mechanism of action translates into clinically meaningful advantages over existing non-surgical modalities (e.g., greater durability, faster onset, or better safety) remains an open question that can only be answered through head-to-head comparative trials.

Several additional limitations of the current evidence base warrant emphasis. First, the lack of standardized diagnostic criteria, severity grading systems, and outcome measures for VL across studies makes inter-study comparisons unreliable (39). Second, no consensus exists regarding optimal treatment parameters for rhCol III, including dosage, frequency, and treatment duration. Third, a substantial proportion of the existing literature on collagen biomaterials exhibits promotional bias, with studies overemphasizing advantages while insufficiently addressing limitations or conducting critical comparative analyses. Fourth, basic mechanistic investigations remain at an early stage, with core molecular targets yet to be definitively validated in vaginal tissue.

## 6 Integrative discussion and future directions

The preceding sections have detailed the collagen-mediated pathophysiology of VL, the material properties and mechanisms of action of rhCol III freeze-dried fibers, and the current state of clinical and preclinical evidence. Synthesizing across these domains, several overarching observations emerge.

First, the shared collagen-based pathophysiology between VL and other PFD subtypes—particularly SUI and POP—provides a strong theoretical rationale for evaluating collagen biomaterials across the pelvic floor spectrum, yet it is critical to emphasize that clinical evidence for rhCol III currently exists only for VL; extrapolation to other PFD conditions remains speculative pending dedicated trials (41). Second, the dual mechanism of rhCol III fibers—immediate physical support combined with active biological regeneration—differentiates this approach from both passive fillers and purely thermal energy-based therapies, but whether this mechanistic distinction translates into clinically meaningful advantages in durability or magnitude of effect remains an open empirical question. Third, the safety profile, while favorable in early studies, requires long-term characterization, particularly given the unique immunological and microbial environment of the vagina.

Looking ahead, several research priorities stand out. Large-scale, multicenter randomized controlled trials with long-term follow-up are needed to establish robust efficacy and safety data. Head-to-head comparative studies against established treatments (e.g., radiofrequency, laser therapy) would define the relative position of rhCol III within the VL treatment algorithm. Standardized diagnostic criteria, severity grading systems, and outcome measures for VL—currently lacking across the field—would greatly improve inter-study comparability. Dose-finding studies and pharmacokinetic profiling are needed to optimize treatment parameters. Finally, investigation of rhCol III for broader PFD indications, particularly SUI and POP, should proceed cautiously from preclinical models through to carefully designed clinical trials.

## 7 Conclusions

Recombinant humanized type III collagen freeze-dried fibers represent an emerging biomaterial-based intervention for vaginal laxity, characterized by high biocompatibility, low immunogenicity, intrinsic collagen regenerative capacity, and minimally invasive delivery. The therapeutic effect is mediated through a tripartite mechanism—physical scaffolding, biochemical regeneration, and microenvironment modulation—although the evidence base for specific molecular pathways derives partially from non-vaginal tissue models and requires direct validation in vaginal tissue. Available clinical evidence from a single small randomized trial demonstrates significant short-term efficacy at 4 weeks, but the evidence base remains insufficient to support rhCol III freeze-dried fibers as an established intervention.

Key knowledge gaps include: the absence of large-scale multicenter trials with long-term follow-up; a lack of standardized diagnostic criteria, treatment protocols, and outcome measures for VL; the need for head-to-head comparisons with established therapies; limited long-term safety data; and uncertain generalizability to other populations. The potential applicability of this approach to broader pelvic floor disorders such as SUI and POP, while biologically plausible, remains entirely speculative and requires dedicated investigation.

Future research should prioritize well-designed clinical trials with standardized endpoints, comparative effectiveness studies, and mechanistic investigations to rigorously validate both therapeutic efficacy and safety. Addressing these gaps will be essential to determine the true clinical value of rhCol III freeze-dried fibers and to define its role within the evolving landscape of vaginal laxity and pelvic floor dysfunction management.
